# Ibuprofen inhibited migration of skeletal muscle cells in association with downregulation of p130cas and CrkII expressions

**DOI:** 10.1186/s13395-019-0208-z

**Published:** 2019-08-29

**Authors:** Chih-Hao Liao, Li-Ping Lin, Tung-Yang Yu, Chih-Chin Hsu, Jong-Hwei S. Pang, Wen-Chung Tsai

**Affiliations:** 1 Department of Physical Medicine and Rehabilitation, Chang Gung Memorial Hospital, No.123, Dinghu Rd., Guishan Dist, Taoyuan City, 333 Taiwan; 2 Graduate Institute of Clinical Medical Sciences, Chang Gung University, Taoyuan City, Taiwan; 3 College of Medicine, Chang Gung University, Taoyuan City, Taiwan; 4 Department of Physical Medicine and Rehabilitation, Chang Gung Memorial Hospital, Keelung, Taiwan

**Keywords:** Ibuprofen, Skeletal muscle, Cell migration, Sport injury

## Abstract

**Background:**

Nonsteroidal anti-inflammatory drugs (NSAIDs) are commonly used to treat sports-related muscle injuries. However, NSAIDs were recently shown to impede the muscle healing process after acute injury. Migration of skeletal muscle cells is a crucial step during the muscle healing process. The present study was performed to investigate the effect and molecular mechanisms of action of ibuprofen, a commonly used NSAID, on the migration of skeletal muscle cells.

**Methods:**

Skeletal muscle cells isolated from the gastrocnemius muscle of Sprague-Dawley rats were treated with ibuprofen. MTT assay (3-[4,5-dimethylthiazol-2-yl]-2,5-diphenyltetrazolium bromide) was used to evaluate cell viability, and cell apoptosis was evaluated by TUNEL assay, after ibuprofen treatment. Skeletal muscle cell migration and spreading were evaluated using the transwell filter migration assay and F-actin staining, respectively. The protein expression of p130cas and CrkII, which are cell migration facilitating genes, was determined by western blot analysis. The overexpression of p130cas of muscle cells was achieved by p130cas vector transfection.

**Results:**

The results demonstrated that ibuprofen did not have a significant negative effect on cell viability and apoptosis. Ibuprofen inhibited the migration and spreading of skeletal muscle cells in a dose-dependent manner. Ibuprofen also dose-dependently decreased the protein expression of p130cas and CrkII. Furthermore, overexpression of p130cas resulted in the promotion of cell migration and spreading and counteracted ibuprofen-mediated inhibition.

**Conclusion:**

This study suggested that ibuprofen exerts a potentially adverse effect on the migration of skeletal muscle cells by downregulating protein expression of p130cas and CrkII. These results indicate a possible mechanism underlying the possible negative effect of NSAIDs on muscle regeneration.

## Introduction

Muscle injuries are one of the most common injuries in sports, and the incidence ranges from 10 to 55% of all injuries [1, 2]. In addition, 37% of male professional football players miss training or competition due to muscle injuries [2, 3]. The current conservative treatment includes limiting the bleeding with compression, elevation, local cooling, nonsteroidal anti-inflammatory drugs (NSAIDs), and physical therapy [1, 4]. NSAIDs are primarily used for their analgesic, anti-inflammatory, and antipyretic properties [5]. The mechanism of action of NSAIDs is cyclo-oxygenase inhibition, which decreases prostaglandin production from arachidonic acid. Decreased prostaglandin level limits the cascading inflammatory response and edema after injury [6, 7]. However, there is a concern that NSAIDs may impede the muscle healing process after acute injury. Piroxicam was shown to delay degradation of damaged tissue and attenuate muscle regeneration in a rabbit muscle strain injury model [8]. Flurbiprofen caused a deficit in torque and force generation of muscles [9]. Besides, NS-398, a COX-2-specific inhibitor, decreased the regeneration of injured muscle by delaying the maturation of regenerating myofibers [10]. A recent study has shown that prostaglandin E2 (PGE2) promoted proliferation of muscle stem cells and NSAIDs that inhibit PGE2 synthesis, may impair muscle regeneration, and lead to weakened muscles [11]. Therefore, NSAIDs might have a potentially harmful effect on the muscle healing process after acute injury.

Satellite cells are myogenic precursor cells in adult skeletal muscles [12]. These are located outside the myofiber plasma membrane and beneath the surrounding basal lamina. Satellite cells play a vital role in muscle regeneration that involves several sequential, but overlapping, stages [12, 13]. Upon severe muscle injury, muscle degeneration initiates myofiber necrosis, leading to increase in myofiber permeability [14]. Calcium influx from extracellular space is associated with nNOS activation [15, 16]. Increased NO production by skeletal muscle results in the release of metalloproteinases, which then mediate the release of wound hormones (e.g., FGF, IGF-1, and HGF) and the activation of satellite cells immediately following severe muscle injury [17–19]. Moreover, myofiber necrosis also activates inflammatory responses [20] that recruit circulating leukocytes to the damage site [21]. After early neutrophil infiltration, macrophages are responsible for the phagocytosis of necrotic debris and also facilitate the proliferation of satellite cells [12, 22, 23]. Muscle injury activates not only the neighboring satellite cells, but also every satellite cell along the same myofiber, which then migrate to the regeneration site even from distant sites [12, 24]. After migration to the site of injury, activated satellite cells proliferate to produce sufficient number of myoblasts for regeneration, commit to differentiation [25, 26], and then form new multinucleated myotubes by cell fusion [27].

Both CT10 regulator of kinase II (CrkII) and p130cas (Crk-associated substrate) are critical for cell motility [28, 29]. CrkII, a member of the Crk adaptor family, was originally isolated as an oncogene product of the avian sarcoma virus, v-Crk [30]. p130cas, also known as breast cancer anti-estrogen resistance 1, is a Crk-associated substrate (Cas) protein family member [31]. p130cas and CrkII have been found with multiple domains and localized with other focal adhesion-associated proteins, such as Src, FAK, and paxillin [28, 32]. In the early spreading, p130cas are phosphorylated by integrin-mediated recruitment of FAK-Src complex at cell-matrix adhesion [33, 34]. Subsequently, p130cas promotes the activation of Ras-related C3 botulinum toxin substrate 1 (Rac1) through interaction with the adaptor protein Crk and stimulates the formation of membrane protrusion [34–38]. Previous studies have shown that blocking p130cas from focal adhesion causes reduced lamellipodia formation and vascular smooth muscle cell migration [39] and that overexpression of CrkII increases cell migration of tongue squamous cell carcinoma cells [40]. Thus, it is possible that inhibition of p130cas and CrkII impairs migration and spreading of skeletal muscle cells. However, these issues have never been investigated and reported.

The hypothesis of this study is that inhibition of skeletal muscle cell migration by ibuprofen is associated with downregulation of p130cas and CrkII expressions. Thus, we aimed to investigate the effects and molecular mechanism of ibuprofen, a commonly used NSAID, on the migration of skeletal muscle cells.

## Method and material

All experimental procedures were approved by the Institutional Animal Care and Use Committee of Chang Gung University (CGU106-042).

### Primary culture of rat gastrocnemius muscle cells

The primary culture method is on the basis of our previous study [41]. The gastrocnemius muscle was obtained from Sprague-Dawley rats (weighing 200 to 250 g, which were provided by BioLasco Taiwan Co. Ltd.), and each muscle was cut into small pieces of approximately 1.5–2.0 mm^3^. With 0.2% collagenase type I in TESCA buffer (50 mM TES, 0.36 mM CaCl_2_) (Sigma-Aldrich, St. Louis, MO, USA) on the cultural plates, these cells were incubated for 45 min at 37 °C in a humidified atmosphere of 5% CO_2_/95% air and then were incubated for another 45 min after being treated with 0.25% trypsin-EDTA (Gibco, Thermo Fisher Scientific, Waltham, MA, USA). The supernatant was collected and undergoes centrifugation with 1000×*g* for 5 min. Cell pellets were re-suspended with Dulbecco’s modified Eagle’s medium (DMEM) (Gibco, Thermo Fisher Scientific, Waltham, MA, USA), with 10% fetal bovine serum (FBS) (Gibco, Thermo Fisher Scientific, Waltham, MA, USA), 5% chick embryo extract(Gibco, Thermo Fisher Scientific, Waltham, MA, USA), 100 U/ml penicillin, and 100 g/ml streptomycin (Gibco, Thermo Fisher Scientific, Waltham, MA, USA). After 1 h for fibroblast-shaped cells adhering to the plate, the non-adherent cells were transferred to another plate for further sub-culture and were incubated at 37 °C in a humidified atmosphere of 5% CO_2_/95% air. Following incubation for 24 h, the supernatant containing skeletal muscle cells were collected into a 15-ml centrifuge tube, cultured in DMEM with 10% FBS, 5% chick embryo extract, and then were centrifuged with 1000×*g* for 5 min. Subsequently, these cells were re-suspended and cultured in a 10-cm culture plate with DMEM with 10% FBS, 5% chick embryo extract, and these cells were used for the following experiment.

### In vitro wound healing model

Skeletal muscle cells were grown on plastic dishes in DMEM with 20% FBS and treated with ibuprofen at different concentrations (0.05 mg/ml, 0.1 mg/ml, 0.2 mg/ml, 0.4 mg/ml, and control) for 24 h. The monolayer of skeletal muscle cells was scraped with a sterile pipette tip to consistently produce a linear cell-free zone (1 mm in diameter) on plastic dishes, and skeletal muscle cells began to outgrow and migrated into the cell-free zone. This method was considered as the process of in vitro healing model. The cell-free zone was photographed at 0 and 12 h after treatment, and the width of the cell-free zone was separately quantified by Image-Pro Premier software (Media Cybernetics, Rockville, MD, USA), and then compared with the initial width at 0 h. Relative wound healing rate was calculated as the ratio of the remaining width of the cell-free zone at 12 h to the original width at 0 h. This experiment was performed in triplicate (*n* = 3).

### Cell viability test

Skeletal muscle cells were treated with ibuprofen at different concentrations (0.05 mg/ml, 0.1 mg/ml, 0.2 mg/ml, 0.4 mg/ml, and control) for 24 h, and the cell viability was measured by MTT test (3-[4,5-dimethylthiazol-2-yl]-2,5-diphenyltetrazolium bromide) (Sigma-Aldrich, St. Louis, MI, USA). MTT reagent (50 μg/ml) was added and incubated at 37 °C for 1 h. The MTT solution was discarded, and 0.5 ml dimethyl sulfoxide (DMSO) was added to dissolve formazan crystals. Aliquots were transferred to the plate of 96 well and detected immediately at 595 nm in a multi-well spectrophotometer, VICTORTM X3 (PerkinElmer Inc., Waltham, MA, USA). This experiment was performed in triplicate (*n* = 3).

### Terminal deoxynucleotidyl transferase dUTP nick end labeling (TUNEL) assay

Skeletal muscle cells were treated with ibuprofen at different concentrations (0.05 mg/ml, 0.1 mg/ml, 0.2 mg/ml, 0.4 mg/ml, and control) for 24 h, and the apoptotic cells were detected by TUNEL assay. We used ApopTag® Fluorescein In Situ Apoptosis Detection Kit S7110 (Merck Millipore, Darmstadt, Germany) to determine apoptotic cells. The apoptotic cells were stained in fluorescein isothiocyanate (FITC) (green), and the nuclei were stained by 4′,6-diamidino-2-phenylindole (DAPI) (blue). The micrographs were obtained at × 200 magnification. The cells were treated with DNase I (3000 U/ml) for 10 min at room temperature as the positive control.

### Transwell filter migration assay

Skeletal muscle cells were treated with ibuprofen at different concentrations (0.05 mg/ml, 0.1 mg/ml, 0.2 mg/ml, 0.4 mg/ml, and control) for 24 h, and the cells were seeded at a density of 1 × 10^5^ cells per filter. Transwell filters (Costar, Corning, Cambridge, MA, USA) with 8.0-μm pores were used for the migration assay. The inner chamber was filled with 200 μl serum-free DMEM, and the outer chamber was filled with 600 μl DMEM with 20% FBS. Cells were allowed to migrate for 3 h at 37 °C in an atmosphere of 95% air/5% CO_2_. The cells were stained with Liu’s stain and then washed twice in PBS. Cells on the upper surface of the filter were removed using a cotton swab. Cells on the lower surface of the filter were counted under eight random fields (HPF) (× 200) per filter and the mean number of migrating cells calculated for each concentration. The experiment was performed in triplicate (*n* = 3).

### Cell spreading assay and immunofluorescence staining

Skeletal muscle cells were treated with ibuprofen at different concentrations (0.05 mg/ml, 0.1 mg/ml, 0.2 mg/ml, 0.4 mg/ml, and control) for 24 h. Then, cells were subcultured and plated on culture dishes with DMEM containing 20% FBS to induce the cell adhesion and cell spreading. After plating for 30 min, cells were fixed in 10% formalin for 15 min, following washing three times in PBS. Cells were permeabilized by 0.1% Triton-X100 in PBS for 5 min. After washing three times in PBS, cells were incubated in blocking solution (3% BSA in PBS) at room temperature for 30 min and incubated for 1 h with phalloidin-conjugated FITC (Sigma, St. Louis, MO, USA) diluted in blocking solution. After washed in PBS, cells were stained in PBS containing 1 μg/ml DAPI for 5 min. After being washed in PBS, the cells were examined under ZOE™ Fluorescent Cell Imager (× 175) (Bio-Rad, Hercules, CA, USA). Three fields were randomly selected for observation and calculation. This experiment was performed in triplicate (*n* = 3).

### Western blot analysis

Cell extracts were prepared in a lysis buffer containing 20 mM HEPES, 1 mM EDTA, 1 mM EGTA, 20 mM NaF, 1 mM Na_3_VO_4_, 1 mM Na_2_P_2_O_7_, 1 mM DTT, 0.5 mM PMSF, 1 μg/ml leupeptin, and 1% Triton X-100. The protein concentration of the cell extracts was determined by Bradford assay (Bio-Rad Laboratories, Richmond, CA, USA). Samples with identical protein quantities were separated by 10% sodium dodecyl sulfate-polyacrylamide gel electrophoresis and transferred onto a PVDF membrane. The membranes were incubated at room temperature in blocking solution (5% BSA in TBST) for 1 h, followed by 2-h incubation in blocking solution containing an appropriate dilution of primary antibody, e.g., GAPDH (Proteintech Group, Inc. Rosemont, IL, USA), anti-phospho-p130cas(Y249), anti-p130cas, anti-CrkII (Cell Signaling Technology, Danvers, MA, USA). After washing, the membranes were incubated in TBS containing anti-mouse IgG conjugated with horseradish peroxidase (Leinco Technologies, Inc., St. Louis, MI, USA) or anti-rabbit IgG conjugated with horseradish peroxidase (Cell Signaling Technology, Danvers, MA, USA) for 1 h. The membranes were washed three times in TBST and developed with Luminata Crescendo Western HRP substrate (Merck Millipore, Darmstadt, Germany). The band intensities were analyzed by BioSpectrum 500 automated imaging system (UVP Inc., Upland, CA, USA). GAPDH was used as an internal control. This experiment was performed in triplicate (*n* = 3).

### Overexpression of p130cas

The overexpression vector of p130cas was obtained from GenEZ ORF clone ORa01354 (Genscript, Piscataway, NJ, USA) which was constructed by the pcDNA3.1+/C-(K)-DYK vector. The pcDNA3.1+/C-(K)-DYK empty vector was used as a negative control (mock). The p130cas vector and mock vector were transfected into skeletal muscle cells by GenJet™ In Vitro DNA Transfection Reagent (SignaGen Laboratories, Gaithersburg, MD, USA) according to the manufacturer’s protocol. After 24 h of transfection, the transfected cells were treated by 0.4 mg/ml ibuprofen for 24 h. Western blot analysis was used for assessing p130cas expression. Transwell filter migration assay and spreading assay were used to evaluate cell migration and spreading.

### Statistical analysis

All data were expressed as the mean ± standard error of the mean (S.E.M). All experiments were performed in triplicate (*n* = 3). The Kruskal-Wallis test was used for comparisons between groups. A Mann-Whitney test was used to identify where the difference occurred. The level of statistical significance was set at a *p* value less than 0.05.

## Results

### Ibuprofen impeded in vitro wound healing of skeletal muscle cells

**Fig. 1:**
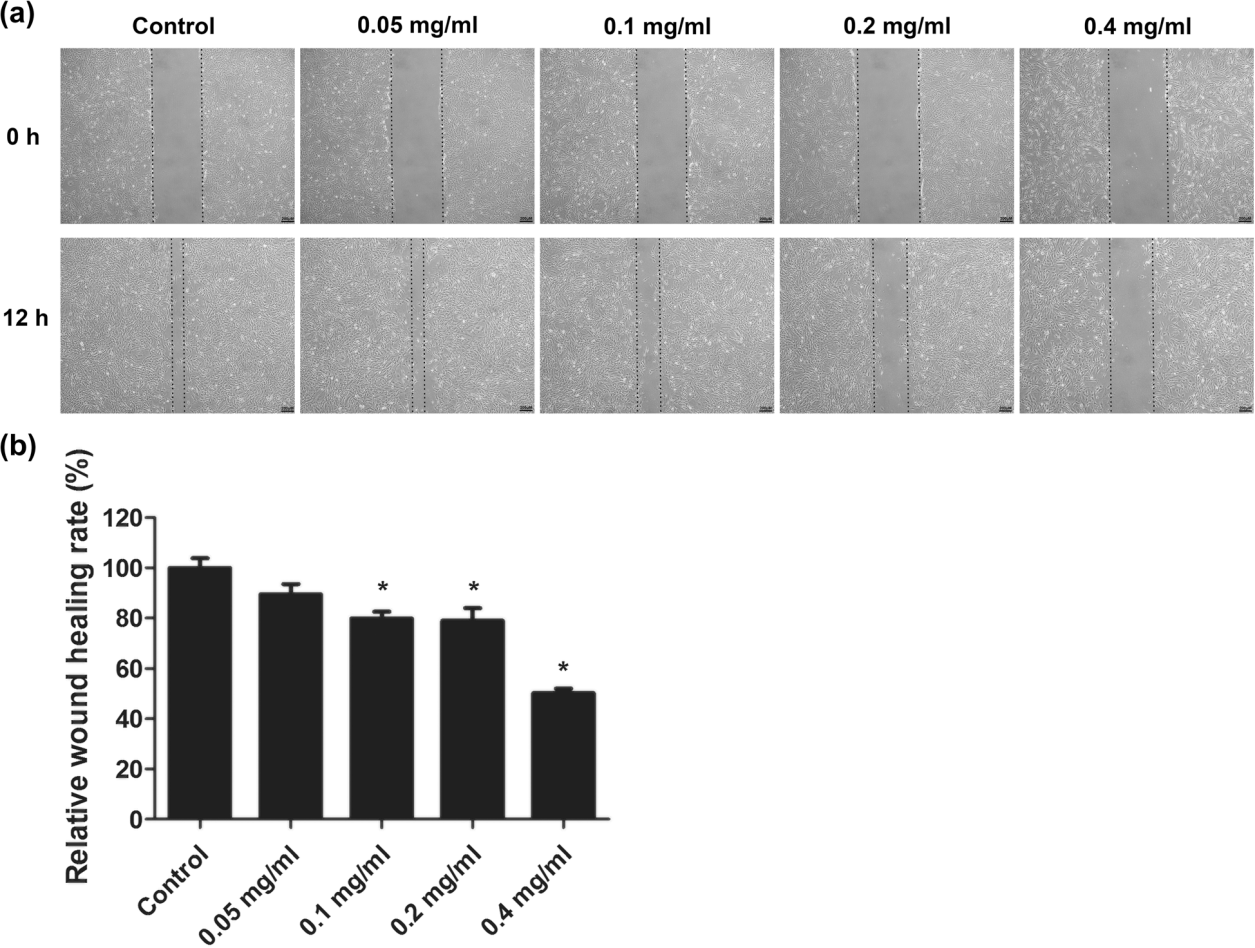
Ibuprofen delayed in vitro wound healing. A monolayer of skeletal muscle cells was treated with 0.05 mg/ml, 0.1 mg/ml, 0.2 mg/ml, and 0.4 mg/ml ibuprofen. After 24 h, the cells were scratched to produce a linear, cell-free zone. The cell-free zones were photographed at × 200 and are indicated by the black dotted lines (**a**). The relative wound healing rate was calculated as the ratio of the remaining width of the cell-free zone at 12 h compared to the original width at 0 h (**b**); the data represented mean ± SEM% of three independent experiments. *Mean *p* < 0.05 compared to the control. Scale bars, 200 μm

To study whether ibuprofen has an inhibitory effect on the skeletal muscle cells, we used in vitro wound healing models. A confluent monolayer of skeletal muscle cells was made and treated with different concentrations of ibuprofen for 24 h. As shown in Fig. 1, the wound healing rate of ibuprofen-treated skeletal muscle cells was slower compared to control cells. The relative wound healing rates were 100.0 ± 3.9%, 89.4 ± 4.1%, 79.8 ± 2.8%, 78.9 ± 5.0%, and 50.2 ± 1.8% in the control, 0.05 mg/ml, 0.1 mg/ml, 0.2 mg/ml, and 0.4 mg/ml ibuprofen-treated cells, respectively. The differences between groups were statistically significant (*p* < 0.05).

### Ibuprofen had no impact on the number of viable skeletal muscle cells

**Fig. 2:**
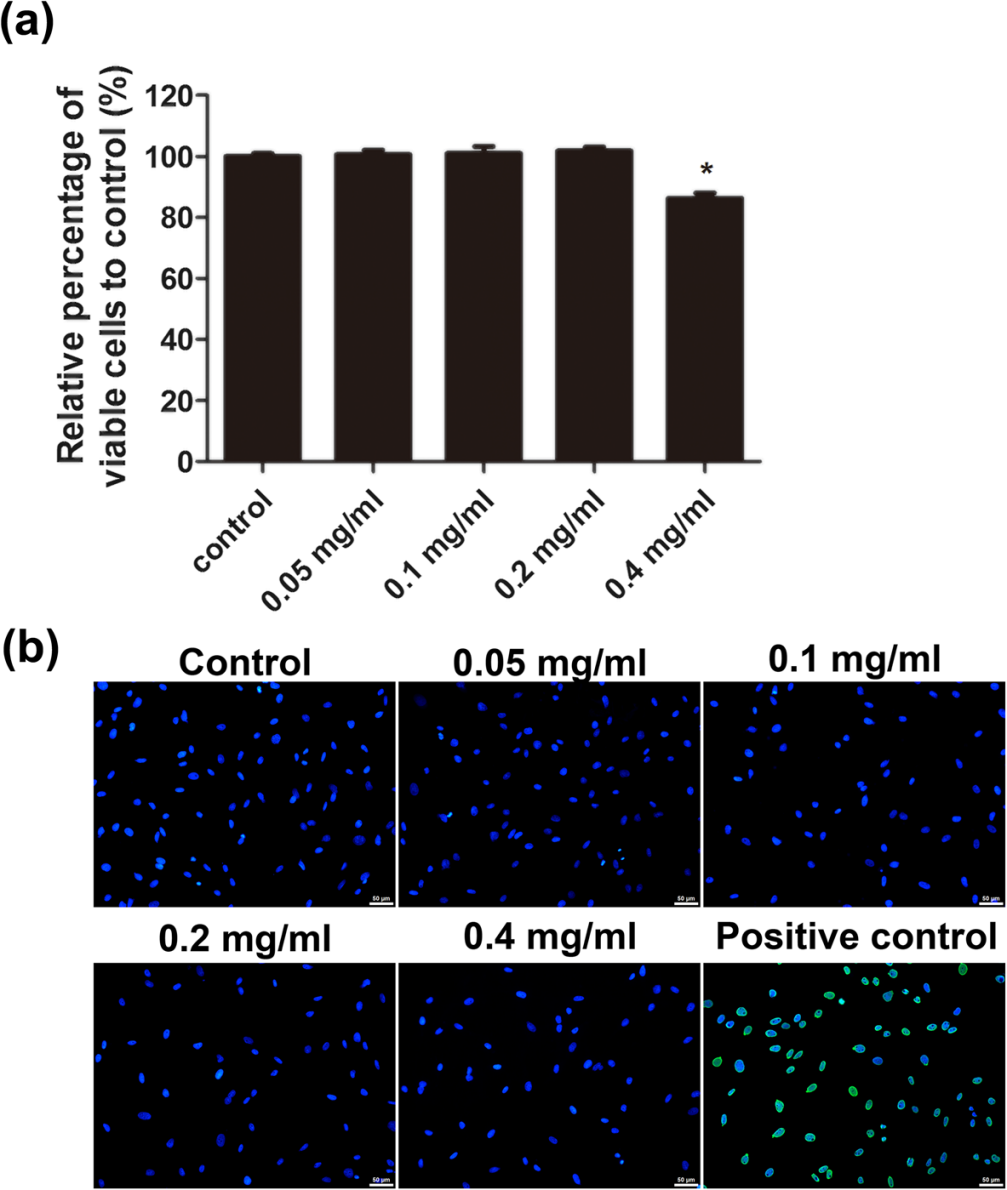
Effects of ibuprofen on cell proliferation and apoptosis. Skeletal muscle cells were treated with 0.05 mg/ml, 0.1 mg/ml, 0.2 mg/ml, and 0.4 mg/ml ibuprofen for 24 h and cell viability was determined by the MTT assay (**a**). The TUNEL-positive nuclei were stained with FITC (green), and all nuclei were stained with DAPI (blue). The micrographs were obtained at × 200 magnification. The cells treated with DNase I were used as positive controls (**b**). Data represented the mean ± SEM of three independent experiments. *Mean *p* < 0.05 compared to the control. Scale bars, 50 μm

In order to investigate cell proliferation, skeletal muscle cells were treated with different concentrations of ibuprofen for 24 h. MTT assay illustrated no significant difference between different concentrations of ibuprofen-treated skeletal muscle cells. However, ibuprofen had a trivial effect on decreasing cell proliferation in at high concentration (0.4 mg/ml). The relative cell counts were 100.0 ± 1.0%, 100.7 ± 1.5%, 101.0 ± 2.3%, 101.9 ± 1.3%, and 86.3 ± 1.7 % in the control, 0.05 mg/ml, 0.1 mg/ml, 0.2 mg/ml, and 0.4 mg/ml ibuprofen-treated cells, respectively (Fig. 2a). Cell death was also examined by TUNEL assay. Apoptotic cells were not detected in all ibuprofen-treated groups (Fig. 2b).

### Ibuprofen inhibited migration of skeletal muscle cells

**Fig. 3:**
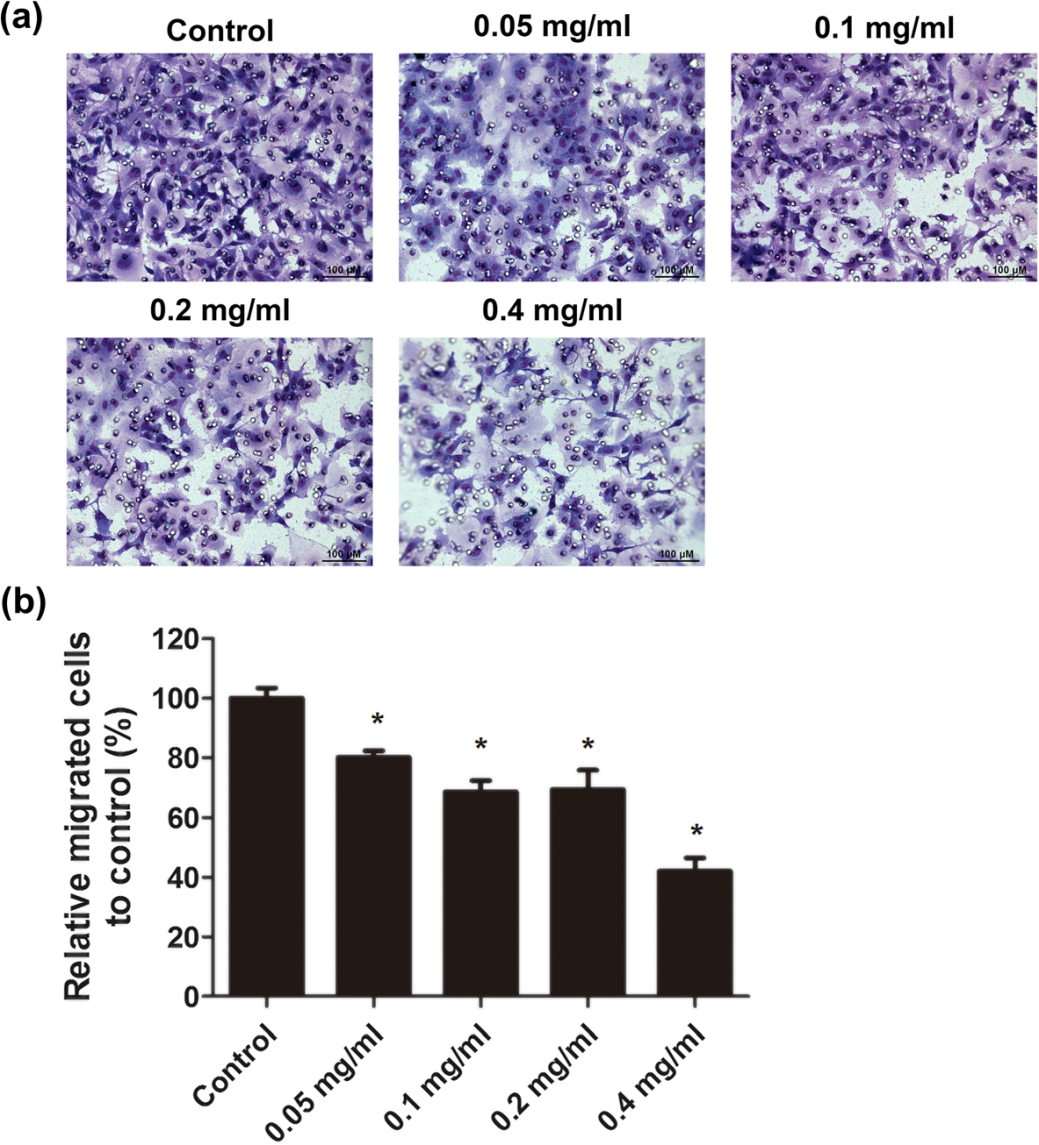
Ibuprofen decreased migration ability of skeletal muscle cells. Skeletal muscle cells were treated with 0.05 mg/ml, 0.1 mg/ml, 0.2 mg/ml, and 0.4 mg/ml ibuprofen for 24 h. The cell migration was assessed by transwell filter migration assay. The cells migrating across the filter were stained by Liu’s stain. The cytoplasm was stained red, and the nucleus was stained blue (**a**). The relative percentage of the migrated cells was shown in **b**, and the data represented the mean ± SEM% of three independent experiments. *Mean *p* < 0.05 compared to the control. Scale bars, 100 μm

Skeletal muscle cells were treated with different concentrations of ibuprofen for 24 h. Migration assay demonstrated that ibuprofen inhibited migration of skeletal muscle cells in a dose-dependent manner. The relative cell migration rates were 100 ± 3.4%, 80.2 ± 2.1%, 68.6 ± 3.8%, 69.3 ± 6.5%, and 42.0 ± 4.5% in the control, 0.05 mg/ml, 0.1 mg/ml, 0.2 mg/ml, and 0.4 mg/ml groups, respectively (*p* < 0.05, Fig. 3).

### Ibuprofen suppressed cell spreading of skeletal muscle cells

**Fig. 4:**
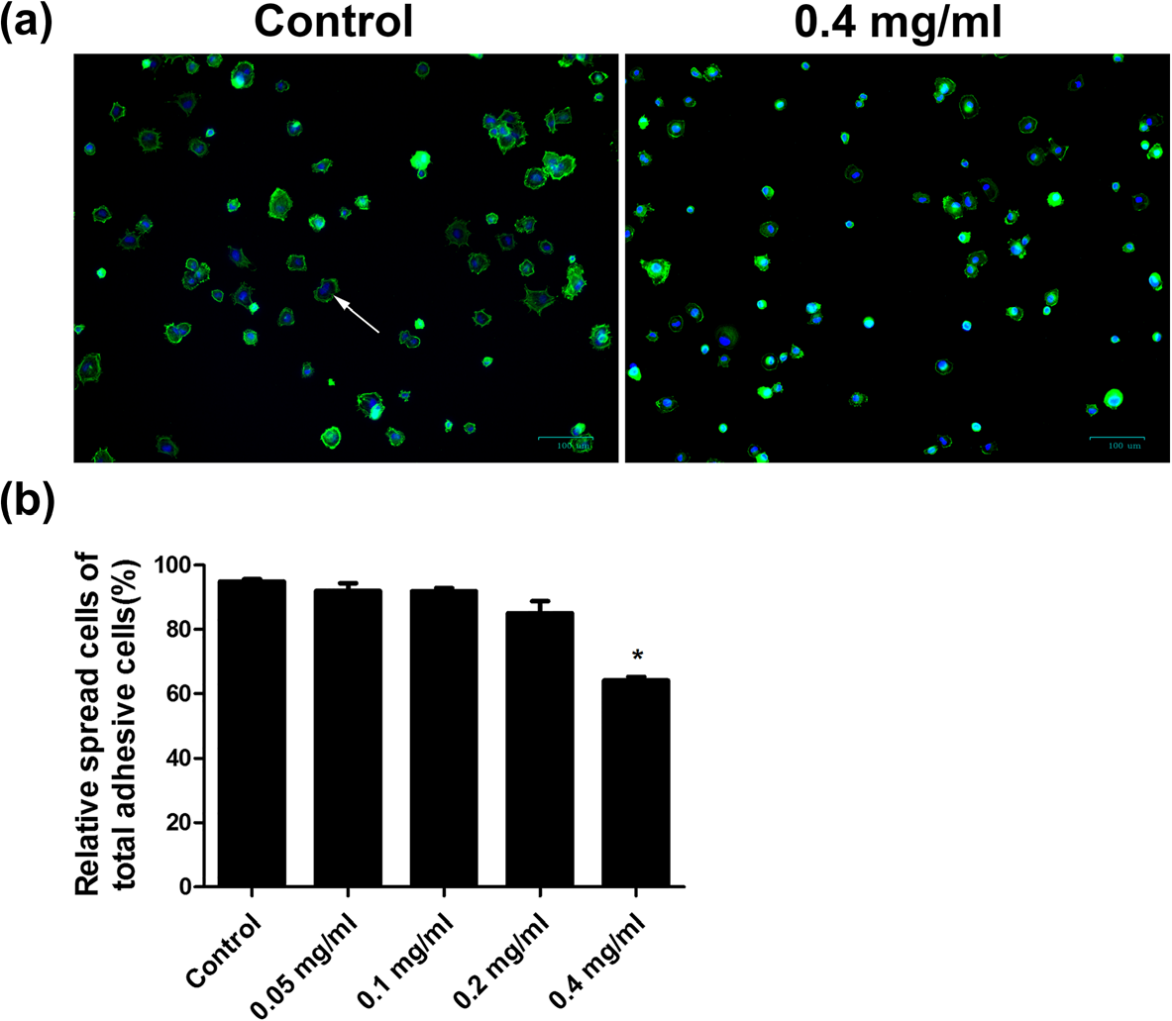
Ibuprofen reduced cell spreading of skeletal muscle cells. Skeletal muscle cells were treated with 0.05 mg/ml, 0.1 mg/ml, 0.2 mg/ml, and 0.4 mg/ml ibuprofen for 24 h. After plating for 30 min, the attached skeletal muscle cells started to spread out and were observed via F-actin staining (**a**). The spread cells were indicated by the white arrows. F-actin was stained green, and nuclei were stained blue. The percentage of spreading cells out of adhered cells was shown in **b**, and the data represented the mean ± SEM% of three independent experiments. *Mean *p* < 0.05 compared to the control. Scale bars, 100 μm

Cell spreading is one of the crucial steps in cell migration. Cell spreading was visualized by performing F-actin staining. The result demonstrated that 0.4 mg/ml ibuprofen reduced cell spreading of skeletal muscle cells compared with controls (Fig. 4a). The relative cell spreading rates were 94.9 ± 0.8%, 91.9 ± 2.5%, 91.8 ± 1.0%, 85.1 ± 3.8%, and 64.1 ± 1.1% in the control, 0.05 mg/ml, 0.1 mg/ml, 0.2 mg/ml, and 0.4 mg/ml groups, respectively (*p* < 0.05, Fig. 4b).

### Ibuprofen downregulated migration-associated protein expression in skeletal muscle cells

**Fig. 5:**
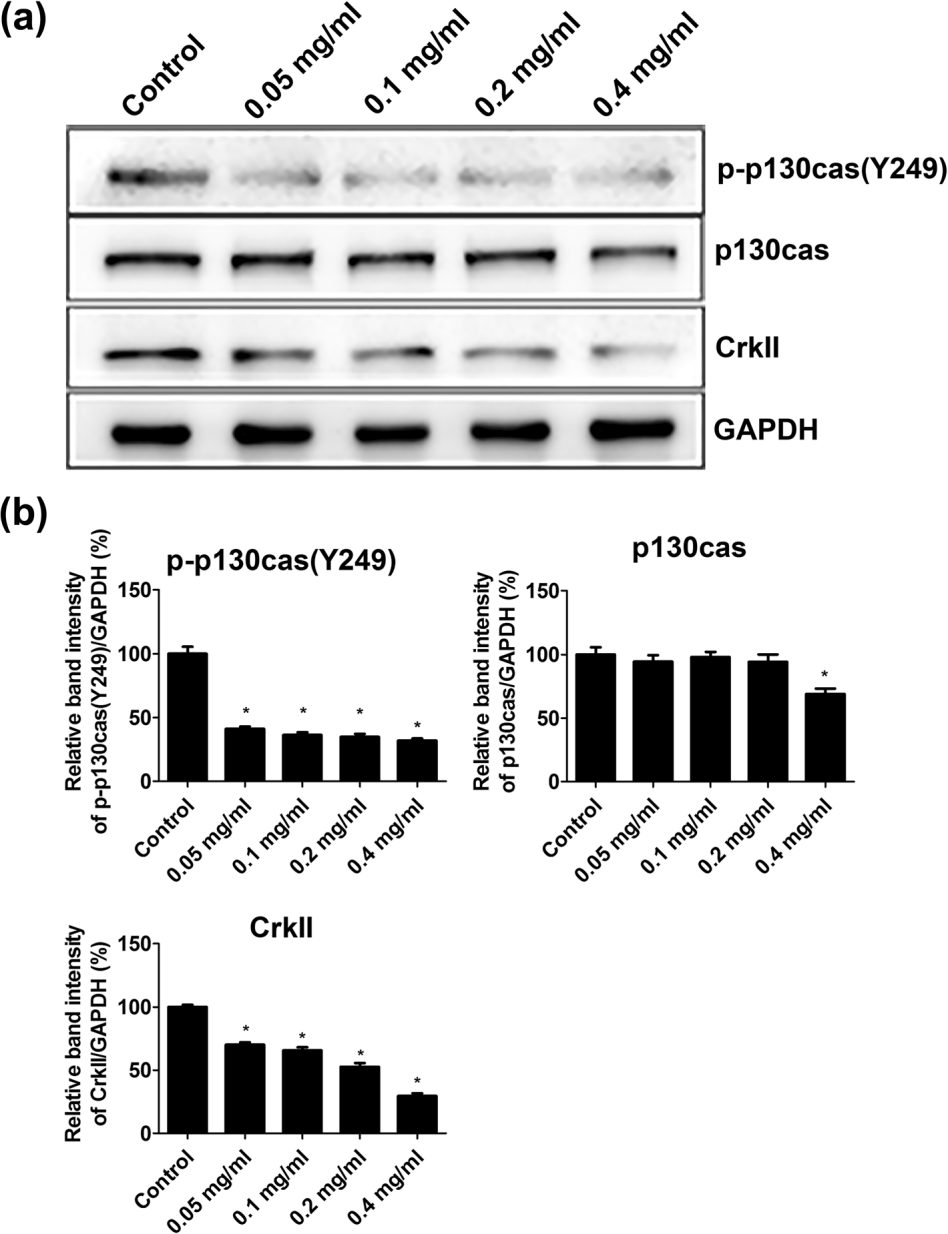
Ibuprofen inhibited the expression of phospho-p130cas, p130cas, and Crk II in skeletal muscle cells. Skeletal muscle cells were treated with 0.05 mg/ml, 0.1 mg/ml, 0.2 mg/ml, and 0.4 mg/ml ibuprofen for 24 h. The protein expression in the cell extracts was analyzed by western blotting (**a**). GAPDH was used as an internal control. The relative band intensities of phospho-p130cas, p130cas, and Crk II were shown in **b**. The data represented the mean ± SEM% of three independent experiments. *Mean *p* < 0.05 compared to the control

Skeletal muscle cells were treated with 0.05 mg/ml, 0.1 mg/ml, 0.2 mg/ml, and 0.4 mg/ml ibuprofen for 24 h. The protein extracts of skeletal muscle cells were analyzed by western blot analysis. Protein expressions of phospho-p130cas, p130cas, and CrkII were downregulated by ibuprofen treatment in a dose-dependent manner. GAPDH was used as an internal control (Fig. 5a). The relative band intensities of phospho-p130cas, p130cas, and CrkII are shown in Fig. 5b.

### Overexpression of p130cas promoted migration and spreading of skeletal muscle cells

**Fig. 6:**
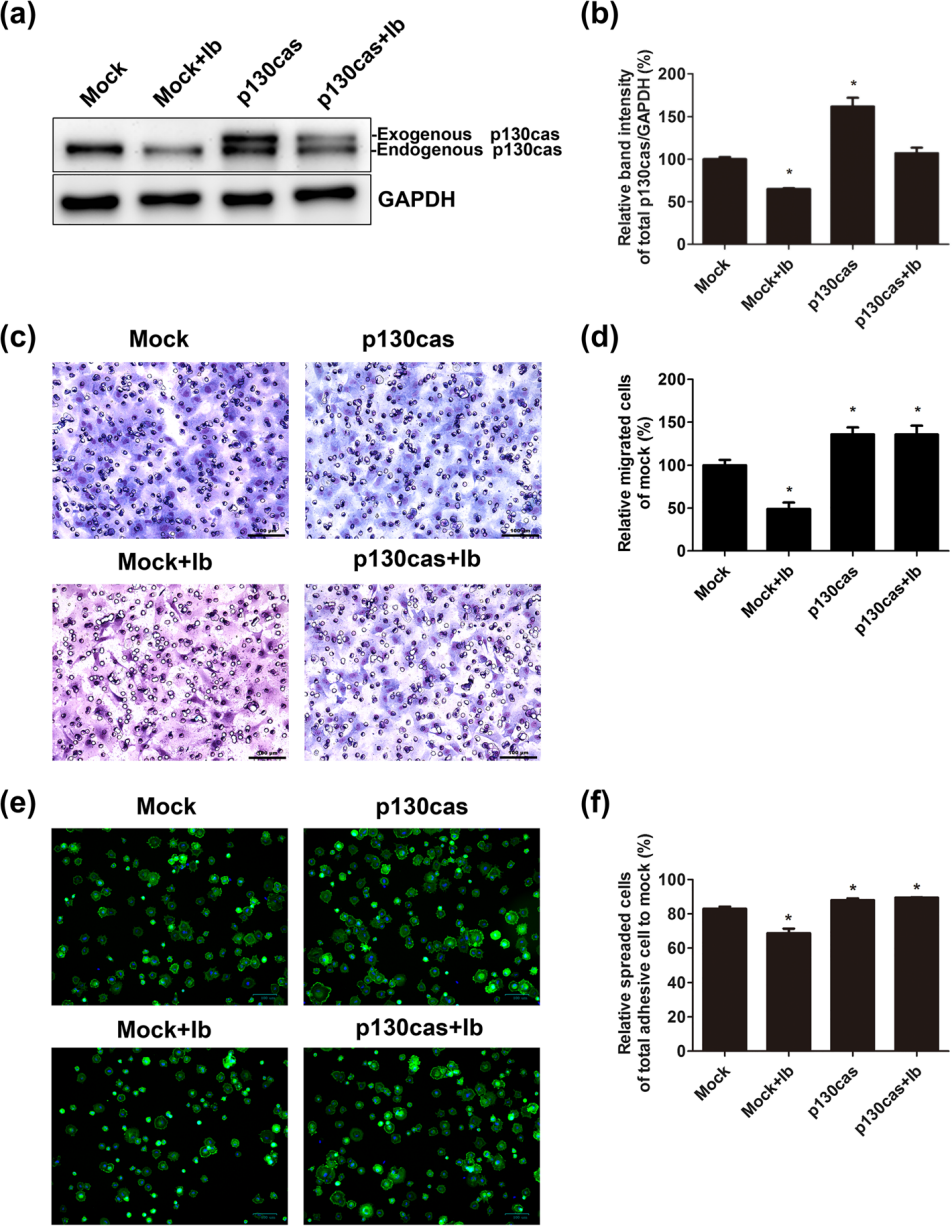
Overexpression of p130cas enhanced cell migration and spreading. Skeletal muscle cells were transfected with a vector expressing p130cas (p130cas) or empty vector (mock). After 24 h of transfection, 0.4 mg/ml ibuprofen was added to the cells transfected with either an empty veotor (mock+Ib) or a vector expressing p130cas (p130cas+Ib) for 24 h. Western blot analysis was used for assessing p130cas expression, and GAPDH was used as an internal control (**a**). The relative band intensities were shown in **b**. Transwell filter migration assay was used to evaluate cell migration ability (**c**), and the relative migrated cell rates were shown in **d**. The cell spreading assay was used to examine cell spreading ability via F-actin staining (**e**) and the relative spread cell rates were shown in **f**. F-actin was stained green, and nuclei were stained blue. The data represented the mean ± SEM% of three independent experiments. *Mean *p* < 0.05 compared to mock. Scale bars, 100 μm

The p130cas vector was transfected into skeletal muscle cells, and the protein expression of p130cas was analyzed by western blot and band intensity analysis. The results revealed that the protein expression of total (endogenous and exogenous) p130cas was significantly upregulated after skeletal muscle cells were transfected with p130cas vector. In p130cas-transfected cells, p130cas expression was comparable to that in mock transfected cells, even after p130cas expression was reduced by ibuprofen (Fig. 6a). The relative band intensities of p130cas were 100.0% ± 2.5%, 64.9% ± 1.0%, 161.8% ± 10.3%, and 107.1% ± 6.6% in the mock transfected cells, mock transfected cells treated with 0.4 mg/ml ibuprofen, p130cas transfected cells, and p130cas transfected cells treated with 0.4 mg/ml ibuprofen, respectively (Fig. 6b). Furthermore, the migration and spreading activities of p130cas transfected cells were increased, and ibuprofen had no effect on the p130cas transfected cells (Fig. 6c, e). The relative migrated cell rates were 100.0 ± 6.4%, 49.1 ± 7.4%, 135.8 ± 8.2%, and 135.6 ± 10.3% in the mock transfected cells, mock transfected cells treated with 0.4 mg/ml ibuprofen, p130cas-transfected cells, and p130cas-transfected cells treated with 0.4 mg/ml ibuprofen, respectively (*p* < 0.05, Fig. 6d). The relative spreading cell rates were 82.9 ± 1.2%, 68.7 ± 2.6%, 87.9 ± 1.1%, and 89.5 ± 0.2% in the mock transfected cells, mock transfected cells treated with 0.4 mg/ml ibuprofen, p130cas-transfected cells, and p130cas-transfected cells treated with 0.4 mg/ml ibuprofen, respectively (*p* < 0.05, Fig. 6f).

## Discussion

Skeletal muscle cell migration is an essential step in muscle regeneration. The healing process consists of three sequential but overlapping phases [12, 13]. In the beginning, muscle fiber necrosis initiates inflammatory response [20]. Macrophages following neutrophils are responsible for phagocytosis of necrosis debris and also facilitate the proliferation of satellite cells [12, 22, 23]. Then, localized muscle injury results in the activation of all satellite cells along the same myofiber. These activated cells then migrate to the injury sites even from the distant sites [12, 24], proliferate to sufficient number of cells, and commit to differentiation [25, 26]. Finally, the newly formed myofibers mature and contract, and the scar tissues are reorganized. Our study demonstrated that ibuprofen impeded migration rather than the proliferation of skeletal muscle cells in a dose-dependent manner, which might indicate a negative effect of NSAIDs for muscle regeneration.

Cell spreading is one of the crucial steps in cell migration. Lamellipodia protrude at the leading edge and new focal adhesions are assembled under the leading edge, and the traction force creates the cell body’s forward movement [42]. NSAIDs were reported to inhibit cell migration and spreading of human umbilical vein of endothelial cells and rat’s Achilles tendon cells [43, 44]. Our results demonstrated that ibuprofen reduced skeletal muscle cell spreading in a dose-dependent manner, which then suppressed cell migration.

This study revealed that ibuprofen inhibited migration and spreading of skeletal muscle cells through downregulation of protein expressions of p130cas and CrkII. In the early phase of cell spreading, the interaction between p130cas and CrkII promotes the activation of Rac1 and subsequently stimulates the formation of membrane protrusion [34–38]. Furthermore, previous studies found that celecoxib-induced inhibition of p130cas contributed to the induction of cell death in colon carcinoma cells [45] and human acute myeloid leukemia cell lines [46]. In addition, it was reported that p130cas silencing impaired actin remodeling and the induction of myogenic differentiation in C_2_C_12_ myoblasts [47]. The phosphorylation of p130Cas, which was driven by integrin β3, was essential for myotube formation [47]. p130Cas plays a major role in response to integrin-mediated stimulation of localized activation of the small GTPase Rac activity [28]. Some studies indicated that NSAIDs inhibited cell spreading and migration by impairing αVβ3 integrin-dependent activation of the small GTPases Cdc42 and Rac [43]. In this study, ibuprofen decreased the protein expressions of both p130cas and CrkII of skeletal muscle cells in a dose-dependent manner. The potential mechanisms of ibuprofen-mediated reduction of p130cas and CrkII may be related to the integrin-dependent pathway.

These findings document the molecular mechanism underlying ibuprofen-induced inhibition of migration and spreading of skeletal muscle cells.

Our findings imply that ibuprofen potentially slowed down the healing process in the skeletal muscle cells. Thus, it is reasonable to postulate that sports activity should be reduced after oral administration of NSAIDs. These findings were compatible with previous studies demonstrating that NSAIDs might impede the muscle healing process after acute injury [8–10]. Furthermore, our study demonstrated that ibuprofen inhibited not only the migration, but also the spreading of skeletal muscle cells. Besides, the downregulation of protein expression of p130cas and CrkII was also found.

We used p130cas vector to overexpress the p130cas expression in skeletal muscle cells, and the result showed that overexpression of p130cas promoted skeletal muscle cell migration and spreading. The results of transwell migration assay and spreading assay revealed that overexpression of p130cas restored the ibuprofen-mediated inhibition. To the best of our knowledge, these findings are novel and have never been reported till now.

The peak plasma concentration of ibuprofen orally absorbed was reported up to 51.3 μg/ml [48], and the concentration of ibuprofen used in our studies was clinically relevant to the dose of oral administration of ibuprofen. In addition, the initial inhibition effect in our study was found at a level of 0.05 mg/ml, which was similar to plasma concentration after oral administration of ibuprofen. These findings indicate that oral administration of NSAIDs for anti-inflammation might have a negative impact on the subsequent muscle healing process. Accordingly, it is imperative to reduce sports activity immediately after muscle injury and also after NSAID use.

## Conclusions

In conclusion, ibuprofen inhibited cell migration and spreading of skeletal muscle cells. The possible underlying molecular mechanisms might be the downregulation of p130cas and CrkII expressions in ibuprofen-treated skeletal muscle cells.

## Data Availability

All data used or analyzed during this study are included in this published article.

## Abbreviations

CrkII: CT10 regulator of kinase II
GAPDH: Glyceraldehyde 3-phosphate dehydrogenase
MTT: 3-[4,5-Dimethylthiazol-2-yl]-2,5-diphenyltetrazolium bromide
NSAIDs: Nonsteroidal anti-inflammatory drugs
p130cas: p130 Crk-associated substrate
TUNEL: Terminal deoxynucleotidyl transferase dUTP nick end labeling
